# Wild elephants vary in their attraction to novelty across an anthropogenic landscape gradient

**DOI:** 10.1098/rsos.250896

**Published:** 2025-07-16

**Authors:** Sarah L. Jacobson, Sangpa Dittakul, Mananya Pla-ard, Supang Sittichok, Marnoch Yindee, Joshua M. Plotnik

**Affiliations:** ^1^ Department of Psychology, Hunter College, City University of New York, New York, USA; ^2^ Department of Psychology, CUNY Graduate Center, New York, USA; ^3^ Golden Triangle Asian Elephant Foundation, Chiang Rai, Thailand; ^4^ Akkhraratchakumari Veterinary College, Walailak University, Nakhon Si Thammarat, Thailand

**Keywords:** personality, neophilia, exploration, Asian elephant, coexistence, human–elephant conflict

## Abstract

Research on how wild animals respond to novelty is becoming more relevant as the overlap between natural habitats and human-dominated landscapes increases. Wild Asian elephants spend more time in anthropogenic landscapes as their habitat is converted to agriculture. Greater neophilia and exploration may allow elephants to successfully access agricultural resources, which may cause negative interactions with people. We compared wild elephant reactions to novel objects in two different landscapes in Thailand (near agriculture and deep inside a protected sanctuary). We also assessed consistency in measures for individuals exposed to different objects to determine whether their reactions could be considered personality traits. Elephants tested near agriculture were more neophilic and exploratory than those inside the sanctuary. However, the limited sample of elephants exposed to both novel objects did not demonstrate consistency in their reactions, and thus we could not determine whether neophilia or exploration were personality traits in this population. Neophilic and exploratory elephants likely benefit from high-quality agricultural resources, but at a potential cost to both elephants and humans. Knowledge about the elephants’ behaviour and attraction to particular landscapes could aid in human–elephant conflict mitigation efforts that consider the needs of both species and aim for more stable coexistence.

## 1. Introduction

Over the past several decades, many researchers have shifted from focusing on the expression of a particular behavioural trait or cognitive capacity in a species to recognizing and quantifying the variation in behaviour between individuals. Many of these differences in behaviour between individuals have been recognized as distinct personality traits when they are consistent across time and context [1]. These consistent differences may explain more than 30% of the phenotypic variation in populations across taxa [2], and many traits are heritable [3–5]. Therefore, this behavioural variation within a population appears to be adaptive in itself and thus is maintained by natural selection; it is not just non-adaptive variation around an ideal behavioural response [6]. Heterogeneity in behaviour within a population may be adaptive within an unpredictable environment as different expressions of personality traits (e.g. bold or shy) can affect fitness depending on the context [1]. For example, when accessing resources, bolder behaviour by an individual in the presence of predators (which incurs significant risk) may be more adaptive when food is limited. However, in an environment where food is more plentiful, a greater fear of predators may reduce the chances of predation on individuals who take fewer unnecessary risks (e.g. [7]). Since individual variation in behaviour clearly has important consequences for fitness, it should be a focus of research about how animals adapt to changing environments.

Specifically, how animals respond to the novelty introduced by anthropogenic impacts—environmental change triggered or influenced by human behaviour—is important for understanding the selective pressures many species face in the wild [8]. While some species have declined due to anthropogenic disturbances [9], others have quickly taken advantage of unexploited ecological opportunities in disturbed or urbanizing environments [10,11]. These environments present many novel challenges to animals, including the need to locate new or known resources in and around human-developed barriers, encountering novel predators and competitors and/or adjusting to anthropogenically driven sensory stimulation from noise, light and chemicals [11]. Knowledge about personality trait variation can contribute to our understanding of how animals adapt to such human-impacted environments and whether certain behavioural patterns lead to negative interactions with humans [12]. Since human-dominated landscapes (HDLs) are typically highly novel compared to the species’ natural habitat, personality traits that describe reactions to novelty are especially relevant for animals in these landscapes [8]. Novelty reactions affect an individual’s exploration of an environment and information gathering, which could influence their survival or reproduction. While investigation of novelty in the environment may lead to new opportunities for food resources, the time spent investigating inedible materials decreases feeding efficiency, and ingesting novel resources could negatively impact the animal’s health. There is also a tradeoff between the benefit of obtaining potential resources and the risk of predation or injury while investigating novelty [13]. Understanding how these tradeoffs affect the decisions of animals can help when predicting how animal behaviour may influence species survival in HDLs.

Reactions to novelty, whether described as neophilia or neophobia, are robust personality traits with strong consistency across many species [14]. However, the relationship between these traits and their expression in different environments is currently debated in the existing literature, which has focused on limited species. One reason for contradictory results may be inconsistent terminology used to describe personality traits [15]. Therefore, we define neophilia as the attraction to novelty based on the intrinsic motivation to approach and investigate it, without another reward present [16]. We are considering this novelty reaction separately from neophobia, or the fear of novelty due to its potential risks [15]. While these two traits may be interrelated, animals can differ in their neophilic and neophobic responses, so they are not necessarily two extremes of a continuum [13]. These traits are probably shaped by different selective pressures depending on ecological conditions, determining whether (i) information about resources is valuable and therefore neophilia is beneficial or (ii) risks of the environment are high, so neophobia is beneficial [13]. In this study, we focus on the trait of neophilia due to its role in motivating initial exploration [15].

Many studies have demonstrated that individuals within a species inhabiting HDLs are more attracted to novelty than those in rural areas [12,17–21]. Also, investigations of invasive species have shown that the individuals colonizing novel landscapes are more neophilic than those in their native range [22,23]. However, other research has found conflicting evidence; for example, there were no differences in how urban mountain chickadees (*Poecile gambeli*) responded to novelty compared to those living in a forest [24], and European blackbirds (*Turdus merula*) were less neophilic in urban than rural areas [25]. After reviewing the comparative literature on neophilia in birds, Griffin *et al*. [26] concluded that whether neophilia was greater in urban or non-urban individuals remained unclear. Expressions of neophilia across different anthropogenic and natural environments may be dependent on a combination of (i) the characteristics of disturbances within a target environment and (ii) the associated ecological challenges faced by the diverse species studied there. Since the majority of studies on how animals respond to novelty have been conducted with avian species [26], more research is clearly needed to understand how different species vary in how they interact with disturbed, anthropogenic landscapes.

The current study focuses on Asian elephants, *Elephas maximus*, to investigate, for the first time, individual variation in neophilic and exploratory behaviour in wild elephants living near HDLs in Thailand. Human activities have decreased the availability of natural habitat for elephants while simultaneously increasing the use of land for agriculture near or encroaching into protected areas (PAs) across Asia [27]. Neophilia may be particularly beneficial to elephants in these agricultural landscapes where the availability of crops for farmers and wildlife alike shifts across time and space based on economic and social decisions related to harvesting time, seasonality or local or national contracts. Neophilia could encourage animals’ exploration of novel areas where human-made objects are prevalent, allowing them to find and access agricultural food resources. However, elephants’ exploration of anthropogenic landscapes and foraging in agricultural fields can lead to large financial losses for farmers and can result in the deaths of both elephants and people [28]. Therefore, understanding the factors that may lead to negative interactions between people and elephants is important for encouraging coexistence between the species [29,30].

Personality has previously been studied in both captive Asian and African savannah elephants (*Loxodonta africana*), primarily using survey methodology where multiple observers or caretakers—who have experience working with the elephants—rate individuals based on defined traits [31–35]. Researchers have also used this survey methodology [36] and have analysed long-term behavioural datasets [37] to characterize the personalities of wild African savannah elephants. However, designating personality traits based on elephants’ behavioural reactions to more objective, experimental conditions has only been conducted in one study with captive elephants. Barrett & Benson-Amram [38] tested Asian and African savannah elephants’ reactions to novelty but did not find that individuals behaved consistently across objects. However, the stimuli used in this study for the assessment of novelty responses were a mixture of novel objects (which are typically used to assess neophilia or neophobia) and predator cues (which are frequently used to assess boldness as a personality trait) [39]. The use of these different types of stimuli, which measure different traits (and thus may not correlate), may have confounded the results. Therefore, we wanted to implement an experimental methodology focused on only novel objects and to assess wild elephants’ responses [40].

In this study, we first compared wild Asian elephants’ responses to two novel objects between the PA of Salakpra Wildlife Sanctuary in western Thailand (an area off-limits to humans other than park rangers and researchers) and the surrounding HDL, where elephant foraging on agriculture is common. Half of the farmers in the area report elephants foraging on their crops daily, and many attempt to guard their fields from elephants every night [41]. When an elephant enters a crop field, farmers will typically use lights, trucks, sound and firecrackers to drive them away, and many people have installed rudimentary electric fences around their fields [41]. We hypothesized that elephants tested near the HDL would be more neophilic and exploratory than those tested deep in the PA, as a tendency to explore novelty would be more advantageous in agricultural areas with artificial constructs. We also hypothesized that neophilia and exploration would vary based on the sex and age class of the elephant. Since males are the dispersing sex and are less likely to be with calves [42,43], we hypothesized that they would be more neophilic and exploratory than females, who are likely to be more risk-averse. In several species, including elephants, younger individuals have demonstrated that they approach and explore novelty more quickly in different experimental contexts (e.g. [44–47]), so we hypothesized that subadults and calves would be more neophilic and exploratory than adults. We also aimed to demonstrate whether neophilia and exploration were consistent personality traits in this population of wild elephants by looking at the elephants’ responses across their exposures to the two different novel objects. We hypothesized that individuals who encountered both types of novel objects would respond consistently towards these objects and that there would be greater variation in responses between individuals than within individuals.

## 2. Methods

### 2.1. Study site

**Figure 1.**
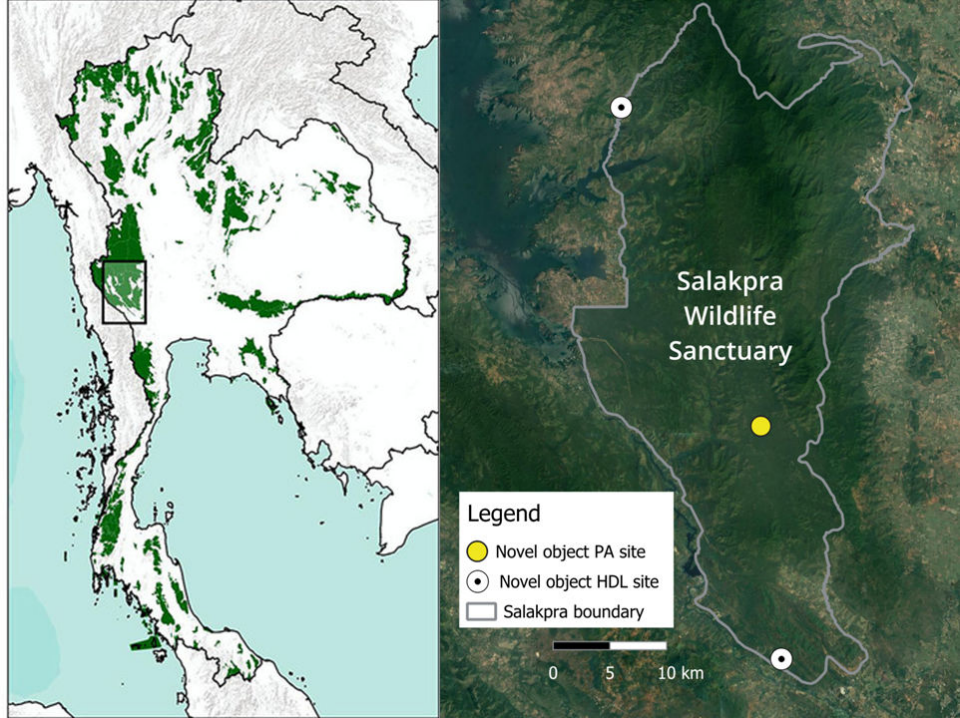
Map of Thailand showing location of Salakpra Wildlife Sanctuary (left image), and site map showing Salakpra and surrounding area with novel object installation sites indicated (right image). Human development can be seen in the brown areas in the satellite image, reflecting the deforested areas around and encroaching into the sanctuary.

Salakpra Wildlife Sanctuary is a PA of approximately 860 km^2^ closed to public tourism. Salakpra is part of a larger complex of forests in western Thailand, which is important for elephant conservation [48]. The landscape of dry dipterocarp forest, mixed deciduous forest and disturbed habitat is home to, as of 2015, an estimated population of more than 250 elephants [49]. Following our development of an individual identification protocol for elephants in Salakpra using video camera traps [50], we now estimate the population to be over 300. There are many villages and agricultural developments bordering Salakpra, with some even encroaching on the official boundaries of the sanctuary (figure 1). Farmers in the area grow sugar cane, cassava, corn and pumpkin. Our experiments were installed at one site near the Khao Seua ranger station, approximately 9 km inside Salakpra (hereafter ‘PA’) where human presence other than rangers is rare, and at two sites within 300 m of agricultural development where human presence is common (Mae Plasoi and Tha Manao villages, hereafter ‘HDL’). Human presence—except for periodic, small party ranger patrols—is rare at the PA site since it is an hour’s drive into the forest with only one accessible road. In contrast, human presence is common in the HDL sites where people frequently cross the ambiguous official boundary to herd livestock and harvest forest products.

### 2.2. Procedure

**Figure 2.**
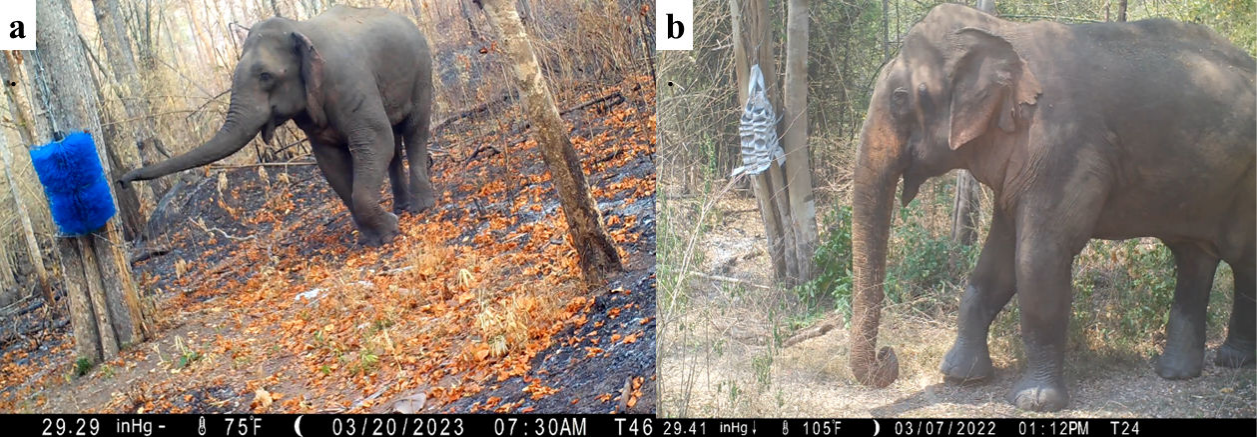
The two novel objects used in neophilia testing: (a) a cattle brush and (b) a woven fire hose object. Still images taken as screenshots from camera trap videos. Photo credit to the Comparative Cognition for Conservation Lab at Hunter College, City University of New York.

The two novel objects chosen to test neophilia were a blue-bristled cattle brush (1 m long and 70 cm in diameter) and a beige woven fire hose object (approximately 1 m × 35 cm × 35 cm; figure 2). These objects were chosen for their size and complexity because greater complexity is likely to elicit novel responses [13,51]. We also chose these objects because they do not resemble forage nor are they likely to be fear-inducing due to any resemblance to a predator or threat [15]. Lastly, we chose objects that were in the colour spectrum that elephants can perceive to enhance their visibility [52]. Neither cattle brushes nor woven firehoses were previously present in the PA or the surrounding areas, so it is extremely unlikely that any elephants have seen these objects before.

At each of the three sites, two identical objects were installed 30−50 m apart to maximize data collection. Each object was secured to a tree using metal slings at a height of approximately 2−3 m (figure 2). The trees were chosen if they were next to an elephant path or appeared to be frequently rubbed against by elephants. Three or four Browning Spec Ops Advantage camera traps were installed on surrounding trees to record the view of a 10 m perimeter around the tree where each object was installed. These cameras recorded 20 s videos when activated by motion and a heat differential, up to 25 m away. The camera traps recorded video using infrared light at night, illuminating 18 m in front of each camera. The 10 m perimeter was determined using a rangefinder, and red, infrared-reflective markers were placed on the ground and on branches to mark the distance. Before the objects were installed, baseline behaviour was recorded for at least two weeks with only cameras and markers present. The cameras and markers remained installed, and videos were recorded between object installations to capture more potential subjects in the baseline condition. The baseline comparisons allowed us to confirm that elephants were behaving differently in response to the novel object itself and not based on some other aspect of that particular location [15].

The experiments were conducted between February 2022 and December 2023. Each of the objects was installed for 34−92 h at a time in each location for 6−10 sessions. The order of object installation was counterbalanced across locations (table 1). We allowed for at least one month between exposures to the different objects; this was longer than the majority of studies assessing novelty reactions reviewed by Takola *et al*. [14]. Due to logistics in scheduling, we were not able to maintain the same time between exposures at each location. Both novel object types were installed on the same trees in all sites except for one installation location that was altered in Khao Seua due to fallen trees. As a result of the infrequent presence of objects on the trees and the length of time between the object installations, it was unlikely that elephants would habituate to the presence of novelty at these sites. The objects were installed for more sessions at some locations when elephant visits were infrequent to increase the potential sample size. The firehose objects were also installed for a second time period at Mae Plasoi to increase the sample of individuals who encountered the objects (table 1). Our research team or a wildlife ranger checked and cleaned the objects between one and three times a day, depending on the location. During each check, the objects were sprayed with 70% isopropyl alcohol, completely wiped clean and dried to ensure that the alcohol completely evaporated (*sensu* [53]). If an object was significantly dirty and could not be wiped clean, it was removed and cleaned more thoroughly off-site, and a fresh object was installed in its place. Our team replaced camera memory cards and batteries for all cameras before installing the objects and after removing them.

**Table 1.** Schedule of novel object installation at each site.

location	object type	dates	time between object installations	shortest time between an elephant’s exposures
Khao Seua (PA)	firehose	21 Feb 2022 to 30 May 2022	1 year	385 days
Khao Seua (PA)	brush	23 May 2023 to 2 Aug 2023		
Tha Manao (HDL)	brush	19 Jun 2022 to 22 Sep 2022	1 month	203 days
Tha Manao (HDL)	firehose	22 Oct 2022 to 13 Apr 2023		
Mae Plasoi (HDL)	firehose	29 Aug 2022 to 12 Oct 2022, 24 Nov 2023 to 20 Dec 2023	2 months, 5 months for second firehose installation	116 days
Mae Plasoi (HDL)	brush	12 Dec 2022 to 28 May 2023		

### 2.3. Coding

Only elephants who entered the 10 m perimeter and who were oriented towards the object were included in the dataset for analysis. Orientation was determined by an elephant’s head facing or trunk reaching towards the object, or, if they were not directly oriented, we included elephants if there was a pause in their movement past the object. We recorded the identities of individual elephants using a combination of morphological traits and our database as described in Montero-De La Torre *et al*. [50]. While some elephants encountered the same object multiple times, we only considered each individual’s first exposure to each object to measure neophilia, since the object would no longer be novel with subsequent exposures (see electronic supplementary material, table S1 for information on all subjects). Using video from all camera angles, we coded elephant behaviour in Behavioral Observation Research Interactive Software [54] following an ethogram to determine each individual’s latency both to approach within a trunk’s length of the object and to touch the object. These measures of initial attraction to novelty have been most typically used to assess neophilia in novel object studies [14]. To assess exploration of novelty, we recorded the time spent (i) within a body’s length of (hereafter ‘in proximity to’) and (ii) interacting with the object, as well as the time spent within the 10 m perimeter (table 2). If an individual never approached or touched the object, they were assigned a maximum latency of 150 s. We also recorded whether the elephant was in a social group or not, based on the presence of another elephant within the 10 m perimeter during the subject’s exposure. We recorded baseline data for individuals’ latencies to approach the installation tree, time spent in proximity to the tree and time spent in the 10 m perimeter in the absence of the novel object if the elephant entered within 10 m of the same tree as their object exposure and prior to their novel object exposure. Most baseline conditions were in the same social context as the novel object exposure; however, in four instances, individuals had a social baseline while their novel object exposure was non-social or vice-versa. The results did not change for baseline comparisons when excluding these individuals, so we included them in the baseline dataset. While other variables may have differed between baseline and object exposure, such as time of day, reproductive status or weather, there were limited opportunities to capture baseline recordings, so we were not able to match every possible variable for these comparisons.

**Table 2.** The ethogram of elephant behaviours coded from videos in both baseline and novel object conditions.

behaviour	condition coded	description
latency to approach	baseline and object	latency starts when elephant steps one foot within 10 m perimeter and ends when within a trunk’s length of the object or object tree
latency to touch	object	latency starts when elephant steps one foot within 10 m perimeter and ends when they contact the object
duration of interaction	object	duration starts from contact with the object and stops after contact ends for at least 5 s, repeated durations are coded
duration spent in proximity	baseline and object	duration starts when elephant steps within one body’s length of the tree or object and ends when the elephant’s back legs are more than a body length away
duration spent in 10 m perimeter	baseline and object	duration starts when elephant steps one foot within 10 m perimeter and ends when back legs leave the 10 m perimeter of the tree
out of view	baseline and object	elephant out of view due to cameras not recording

We set up the camera traps to record almost continuously with a fast recovery speed (0.4 s) between triggers, but sometimes cameras did not record when elephants were within the 10 m perimeter of the objects. Therefore, we also recorded the amount of time in each observation where elephants were out of view due to cameras not recording. Across all observations, including the baseline, elephants were out of view for a median of 4.80% (IQR 0.00%–18.34%) of the observation.

The first author coded all videos, and another student blind to the hypotheses and locations of the videos coded 20% of baseline and novel object videos (34 videos) using the same ethogram. Using intraclass correlation analysis, we found good agreement between coders for latencies to approach the objects (ICC(1)=0.82, *F*(33,34) = 10.20, *p* < 0.001) and excellent agreement between coders for latencies to touch the objects (ICC(1) = 0.90, *F*(24,25) = 18.30, *p* < 0.001) as well as for duration in the 10 m radius (ICC(1) = 1.00, *F*(33,34) = 1024, *p* < 0.001), duration in proximity (ICC(1) = 0.96, *F*(33,34) = 45.10, *p* < 0.001) and duration of interaction with the object (ICC(1) = 0.99, *F*(24,25) = 151, *p* < 0.001).

### 2.4. Analysis

Elephant latencies to approach and touch the novel objects were modelled using Cox proportional hazards models in the ‘survival’ and ‘simPH’ packages [55,56]. We used Cox proportional hazards models for these data because the models can appropriately account for events that did not occur within the observation (i.e. individuals who never approached or touched the objects). This avoids the assignment of arbitrary maximum values in place of absences in the analysis, which could skew the data. The Cox proportional hazards model and its extension including a random effect (a frailty model) account for time-dependent and right-censored data and are increasingly used in behaviour studies [57,58]. We used linear models in the lme4 package [59] to analyse the association of different variables with the time elephants spent in the 10 m perimeter of, in proximity to and interacting with the novel object. To normalize these data, we added 1 s and took the base 10 logarithm of each duration. The assumption of proportionality was checked for Cox proportional hazards models, and all linear model residuals were assessed using the ‘DHARMa’ package [60]. We included all variables outlined for each comparison below in our models in order to account for their effects and did not use a model selection process (see electronic supplementary materials for model details). All analyses were done in R v 4.3.2 [61].

### 2.5. Baseline and human-dominated landscape site comparisons

First, we assessed whether the presence of the novel object affected elephants’ behaviour around the installation tree for the subset of individuals who were observed in both baseline and novel object conditions (*n* = 43 elephants, 86 observations). The fixed effect of condition (baseline versus novel object) was included in separate models of latency to approach the novel object tree, time spent in the 10 m perimeter and time spent in proximity of the novel object tree. Any latencies or durations that were not affected by the presence of the novel object were not included in subsequent analyses. Then, to determine whether we could combine novel object data from elephants tested in the two HDL sites (Tha Manao and Mae Plasoi), we assessed whether elephant behaviour was similar between those sites (*n* = 53 novel object observations). We assessed the fixed effect of site (Tha Manao versus Mae Plasoi) on latency and duration measures and then pooled data from the two sites into one HDL variable because there was no effect.

### 2.6. Location comparison

We assessed whether the location (PA versus HDL) affected latencies to approach and touch the novel objects, as well as the time spent in proximity to and interacting with the objects in each elephant’s first exposure (*n* = 125 observations). In these models, we also included fixed effects of object type (brush versus firehose), sex and age class of the subject. To account for any effects of social context on behaviour, we also included a binary variable categorizing whether other elephants were present in the 10 m perimeter. Because we were only able to clean or replace objects at least once a day, we included another binary variable categorizing whether another elephant had interacted with the object prior to the subject’s exposure and before it had been cleaned.

### 2.7. Consistency in behaviour

Lastly, to determine whether the subset of elephants who were exposed to both objects (*n* = 19 elephants) had consistent reactions between objects, we compared latencies and durations between exposures. For both latency measures, we created frailty models including the random effect of elephant identity. Then, we compared the fit of the frailty models to the Cox proportional hazards models without elephant identity included using likelihood ratio tests. These models also included fixed effects of exposure number (whether they encountered the object first or second) and any significant effects from the first exposure models. We also calculated intraclass correlations to determine repeatability in the elephants’ latency measures between the two objects using the random effect variance from the frailty models, as outlined in McCune *et al*. [57]. To assess consistency for duration measures, we created linear mixed models including fixed effects of exposure number and any variables that had significant effects from the first exposure model, as well as a random effect of elephant identity. The transformed durations were used as the outcome variable. We used the ‘rptR’ package to calculate adjusted repeatability or the proportion of variance attributed to the subject identity in these models while controlling for fixed effects [62].

## 3. Results

### 3.1. Baseline and human-dominated landscape site comparisons

There was a significant effect of condition (baseline versus novel object) on latencies to approach the installation tree for the 43 elephants that experienced both novel object and baseline conditions (novel object: *β* = 1.20 ± 0.36, *p* < 0.001; table 3). There were 31 baseline observations in which the elephant did not approach the tree. For the 12 baseline observations in which the elephant did approach the tree, the mean latency to do so was 62.98 ± 45.93 s (mean ± s.e.). There were 15 observations in which the elephant did not approach when a novel object was present. For the 28 observations in which an elephant approached when a novel object was present, the mean latency to do so was 18.35 ± 2.89 s. There was also a significant effect of condition on duration in proximity to the installation tree for those elephants (novel object: *β* = 1.45 ± 0.37, *p* < 0.001; table 4). In baseline conditions, elephants were in proximity to the tree for a mean duration of 10.39 ± 3.76 s, and in novel object conditions, 72.58 ± 18.89 s. However, when assessing the time the elephants spent within the 10 m perimeter, there was no effect of condition on this measure (baseline: 95.36 ± 21.76 s, novel object: 119.4 ± 20.11 s; table 4). Therefore, we did not use duration in the 10 m perimeter in subsequent analyses because the novel object condition was not influencing this measure.

**Table 3.** Cox proportional hazard model results of the effect of condition (baseline or novel object) on the latency to approach the novel object tree.

fixed effect	estimate	standard error	hazard ratio	*z*	*p*	hazard ratio 95% CIs
condition (novel object)	**1.20**	**0.36**	**3.33**	**3.37**	**<0.001**	**(1.65, 6.71)**

Note. CI = confidence interval. Significant results are bolded (*p* < 0.05), and the reference category that the coefficient for condition is compared to is the baseline condition.

**Table 4.** Linear model results of the effect of condition (baseline or novel object) on durations in proximity and within 10 m of the novel object tree.

model	fixed effect	estimate	standard error	*t*	*p*
duration in proximity	condition (novel object)	**1.45**	**0.37**	**3.94**	**<0.001**
duration in 10 m perimeter	condition (novel object)	0.31	0.21	1.47	0.15

Note. Significant results are bolded (*p* < 0.05), and the reference category that the coefficient for condition is compared to is the baseline condition.

For the assessment of latency to approach and touch the novel object between the two HDL sites, we observed no effect of site on the latencies (electronic supplementary material, table S2). There were 15 observations in which an elephant did not approach the object at the Tha Manao site and 3 observations in which the elephant did not approach at the Mae Plasoi site. For the 22 observations at Tha Manao in which the elephant approached the object, the mean latency to approach was 12.41 ± 1.96 s. For the 13 observations at Mae Plasoi in which the elephant approached the object, the mean latency was 18.75 ± 3.92 s. There were 18 observations in which an elephant did not touch the object at Tha Manao and 9 observations in which an elephant did not touch at Mae Plasoi. For the 19 observations at Tha Manao in which an elephant touched the object, the mean latency to touch was 17.22 ± 2.69 s. For the seven observations at Mae Plasoi in which an elephant touched, the mean latency was 23.75 ± 5.50 s. There also was no effect of site on duration in proximity to (Tha Manao: 78.81 ± 22.08 s, Mae Plasoi: 54.54 ± 32.01 s) or duration of interaction with (Tha Manao: 70.63 ± 20.99 s, Mae Plasoi: 29.39 ± 20.91 s) the novel objects (electronic supplementary material, table S3). Therefore, the two sites were combined into one HDL category for subsequent analyses.

### 3.2. Location comparison

**Figure 3.**
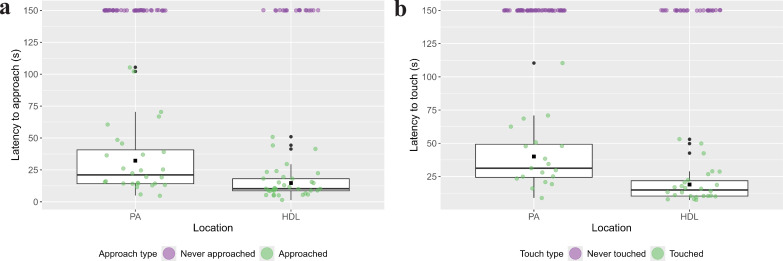
The raw latencies to (a) approach and (b) touch the novel object between elephants tested near HDL and in PA in green points. Box plots represent the median and interquartile intervals of latencies for elephants who did approach and touch, with square, black points representing the means. Raw data point for individuals who did not approach and touch are plotted in purple at the maximum value to which they were assigned and did not contribute to the box plots or means.

Although it was possible that elephants could have moved between locations and been exposed to novel objects in both the PA and HDL, no elephants identified in this study were tested in both locations. Therefore, the samples were treated as independent, and individuals were categorized based on where they were tested (i.e. in the PA or near HDL). Overall, in 63 of the 125 observations (50%), elephants approached within trunk’s length of the object, and in 45 observations (36%), elephants touched the objects. The proportional hazards assumptions were violated for the models of both latency to approach and to touch the objects, such that the effect of location was not constant across latencies. Therefore, an interaction between latency in milliseconds (ms, log-transformed) and location was included to accommodate for nonproportional hazards in both models [56]. There was a significant effect of location on latency to approach (*β* = 11.00 ± 4.11, *p* = 0.007) and touch (*β* = 19.16 ± 6.76, *p* = 0.005) the novel object, with elephants near HDL more likely to approach and touch more quickly than elephants in the PA (table 5). In 35 of the total 53 observations near HDL, the elephants approached the objects with a mean latency of 14.76 ± 1.95 s; in 18 observations, elephants did not approach. In 28 of the total 72 observations in the PA, elephants approached the objects with a mean latency of 32.22 ± 5.08 s; in 44 observations, elephants did not approach. In 26 of the 53 observations near HDL, the elephants touched the objects in 18.98 ± 2.47 s (mean latency); in 27 observations, they did not touch the objects. In 19 of the 72 observations in the PA, elephants touched the objects in 40.03 ± 5.63 s (mean latency); in 53 observations, they did not touch (figure 3). The interactions were also significant in both models, demonstrating that the effect of location decreased as latencies increased (electronic supplementary material, figure S1). No other factors had significant effects on elephants’ latencies.

**Table 5.** Cox proportional hazard model results for the latency (ms) to approach and touch the novel objects.

model	fixed effect	estimate	standard error	hazard ratio	*z*	*p*	hazard ratio 95% CIs
approach	location (HDL)	**11.48**	**4.16**	**9.64 × 10^4^**	**2.76**	**0.006**	**(27.53, 3.38 × 10^8^)**
	object (firehose)	0.38	0.27	1.46	1.40	0.16	(0.86, 2.46)
	sex (male)	0.24	0.47	1.27	0.51	0.61	(0.51, 3.20)
	age class (subadult)	−0.17	0.29	0.84	−0.60	0.55	(0.47, 1.49)
	age class (calf)	−0.77	1.04	0.46	−0.74	0.46	(0.06, 3.57)
	social (yes)	−0.44	0.38	0.65	−1.14	0.26	(0.31, 1.37)
	previous elephant interaction (yes)	0.14	0.35	1.15	0.40	0.69	(0.58, 2.28)
	log(latency) × location	**−1.08**	**0.43**	**0.34**	**−2.54**	**0.01**	**(0.15, 0.78)**
touch	location (HDL)	**19.53**	**6.74**	**3.03 × 10^8^**	**2.90**	**0.004**	**(553.06, 1.67 × 10^14^)**
	object (firehose)	0.46	0.32	1.58	1.41	0.16	(0.84, 2.99)
	sex (male)	−0.25	0.52	0.78	−0.48	0.63	(0.28, 2.17)
	age class (subadult)	−0.28	0.36	0.76	−0.77	0.44	(0.38, 1.53)
	age class (calf)	0.02	1.07	1.02	0.02	0.99	(0.13, 8.22)
	social (yes)	−1.08	0.52	0.34	−2.00	0.05	(0.12, 0.98)
	previous elephant interaction (yes)	0.55	0.38	1.73	1.44	0.15	(0.82, 3.65)
	log(latency) × location	**−1.86**	**0.67**	**0.16**	**−2.78**	**0.005**	**(0.04, 0.58)**

Note. CI = confidence interval. Significant results are bolded (*p* < 0.05), the reference category that the coefficient is compared to for location is PA, object is brush, sex is female, age class is adult, social is no (i.e. not in a social group) and previous elephant interaction is no (i.e. no other elephants interacted with object prior to the subject). In total, 125 observations were included in the dataset; however, the model only analysed 123 observations because sex was unknown for three calves.

**Figure 4.**
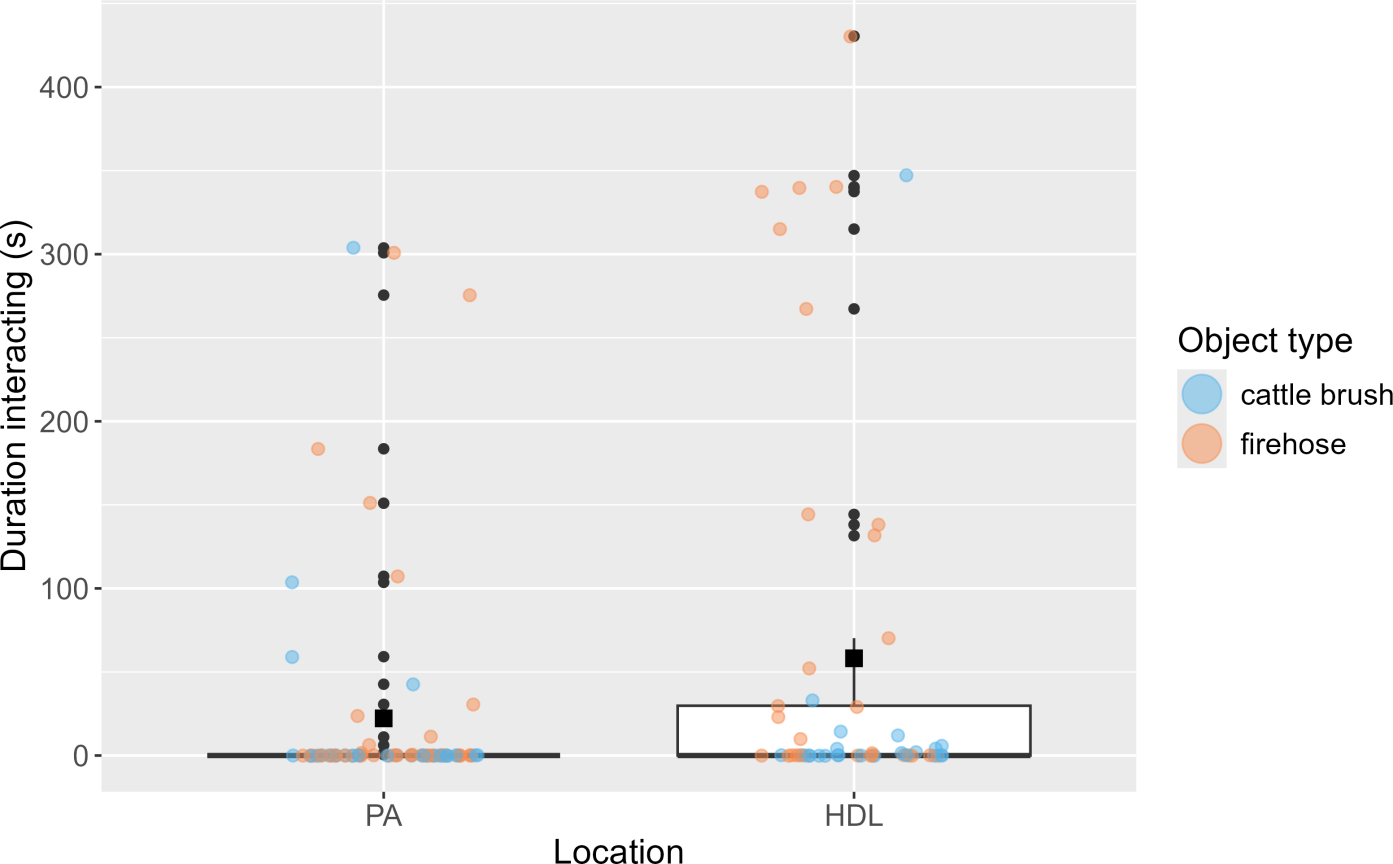
The duration of elephant interactions with the novel objects in seconds in the PA and in the HDL. The medians are represented by the thick black line, and each coloured point represents the raw data for cattle brush objects (blue) and firehose objects (orange). Durations were log-transformed for analysis, but raw data are plotted here for clarity. Black, square points represent the mean duration of interaction for each location.

There was no significant effect of location, but there was a significant effect of object (*β* = 0.92 ± 0.35, *p* = 0.01) on the time elephants spent in proximity to the novel object. Elephants spent more time in proximity to the firehose object (mean ± s.e., 106.76 ± 39.86 s) than the cattle brush object (37.49 ± 13.46 s). There were no other significant effects on time in proximity to the object (table 6). When assessing the time elephants spent interacting with the novel objects, there were significant effects of both location (*β* = 0.90 ± 0.36, *p* = 0.03) and object (*β* = 0.83 ± 0.36, *p* = 0.02). Elephants spent more time interacting with the objects in HDL (92.00 ± 35.06 s) than in the PA (13.61 ± 8.79 s) and more time interacting with the firehose (85.90 ± 34.12 s) than the cattle brush (23.84 ± 18.28 s; figure 4). No other factors significantly affected the durations of interaction with novel objects (table 6).

**Table 6.** Linear model results for duration spent in proximity to and interacting with the novel object. Estimated effects of predictor variables on the log-transformed (duration +1).

model	fixed effect	estimate	standard error	*t*	*p*
duration in proximity	intercept	1.05	0.61	1.71	0.09
	location (HDL)	0.56	0.36	1.57	0.12
	object (firehose)	**0.92**	**0.35**	**2.61**	**0.01**
	sex (male)	0.72	0.57	1.27	0.21
	age class (subadult)	−0.41	0.38	−1.07	0.29
	age class (calf)	−0.007	0.91	0.008	0.99
	social (yes)	−0.72	0.48	−1.49	0.14
	previous elephant interaction (yes)	0.15	0.45	0.32	0.75
duration interacting	intercept	0.43	0.62	0.70	0.48
	location (HDL)	**0.80**	**0.36**	**2.21**	**0.03**
	object (firehose)	**0.83**	**0.36**	**2.32**	**0.02**
	sex (male)	0.27	0.57	0.48	0.63
	age class (subadult)	−0.44	0.39	−1.14	0.26
	age class (calf)	−1.28	0.92	−1.39	0.17
	social (yes)	−0.92	0.48	−1.90	0.06
	previous elephant interaction (yes)	0.75	0.46	1.65	0.10

Note. Significant results are bolded (*p* < 0.05). The reference category that the coefficient is compared to for location is PA, object is brush, sex is female, age class is adult, social is no (i.e. not in a social group) and previous elephant interaction is no (i.e. no other elephants interacted with the object prior to the subject). In total, 125 observations were included in the dataset; however, the model only analysed 123 observations because sex was unknown for three calves.

### 3.3. Consistency in behaviour

There were 19 elephants who were exposed to both the firehose and cattle brush objects. For latency data from these elephants, the frailty models including subject identity did not significantly fit the data better than the models without subject identity (approach: *χ*^2^(1) = 0.45, *p* = 0.50; touch: *χ*^2^(1) = 2.29, *p* = 0.13). The intraclass correlation coefficients were also non-significant and calculated as 0.15, *p* = 0.26 for latency to approach and 0.38, *p* = 0.07 for latency to touch. Latency to approach or touch the novel objects also did not change across exposures (electronic supplementary material, table S4). Therefore, elephant latencies to approach and touch the objects were not affected by whether it was the first or second object they encountered and they were not consistent between the firehose and cattle brush objects.

The duration in proximity (adjusted *R* = 0.25 ± 0.19, *p* = 0.11) and duration of interaction (adjusted *R* = 0.32 ± 0.19, *p* = 0.07) were not significantly repeatable between the two object exposures. Durations in proximity to and interacting with the novel objects were also not affected by exposure number (i.e. whether the firehose object was encountered first or second; electronic supplementary material, table S5). Measures of elephant exploration of the novel objects were therefore not affected by exposure order, nor were consistent between the firehose and cattle brush objects.

## 4. Discussion

Wild elephants tested in the HDL near agriculture were more neophilic and explorative than those tested deep inside the PA. This was supported by their behaviours demonstrating initial attraction to the novel objects; the HDL elephants were more likely to approach within a trunk’s length of the objects and contact them with their trunk or body, also doing so more quickly than PA elephants. These measures of initial attraction are commonly used to establish the personality trait of neophilia [14]. Our assessment of the elephants’ exploration of novel objects showed more inconsistent results. Elephants in the HDL interacted with objects longer than those in the PA, but there was no difference between these locations in the time elephants spent near an object. Instead, only the object type affected the time spent in proximity, with elephants spending more time close to the firehose than the brush. This difference may have resulted from the different properties of the objects’ materials; elephants may have spent more time near the more porous firehose, which could have held scents for longer than the plastic brush bristles, despite our efforts to clean all of the objects thoroughly. Elephants also interacted with the firehose for longer than the cattle brush overall, so there may have been other characteristics of this object that were more attractive to elephants. Even with these differences in exploration between the novel objects, overall, the location at which the elephants were tested was associated with both measures of neophilia and one of the measures of exploration.

These results are consistent with evidence that anthropogenic landscapes promote greater attraction to and exploration of novelty in other species. Spotted hyenas (*Crocuta crocuta*), living at the border of a national park where human and livestock activities were prevalent, were less afraid of novel objects and more exploratory than those living within the relatively undisturbed landscape of the park [12]. In several bird species, individuals living in urban areas were more attracted to or less afraid of novelty compared to conspecifics living in rural areas [26]. While the HDL surrounding Salakpra Wildlife Sanctuary does not contain the same degree of novel human structures as in the city environments that have served as comparison landscapes in many avian studies, there are fluctuating human artefacts in the agricultural landscape and increasing human encroachment into the sanctuary that present wild elephants with novel stimuli. For example, the different equipment brought in for planting or harvesting crops and structures built seasonally for farmers to guard their fields can all be novel for elephants. In contrast to our results, some comparisons in birds have found that individuals in cities are less attracted to novelty than conspecifics in rural landscapes [25,63]. It is possible that there are more risks associated with novel stimuli in a city environment for birds than the risks associated with novel stimuli in the HDL for elephants, therefore favouring increased neophobia in birds. The expression of novelty responses in different species may also be influenced by predation risk [64]; for example, animals that experience less predation, like elephants, may be less risk-averse, which may make them more likely to approach novelty. Neophilia is likely to be more beneficial to species when information about resources is valuable [13], so elephants’ increased attraction to novelty in the HDL indicates that novel information related to obtaining agricultural resources may be more beneficial to elephants than the potential costs of any risks they might face. Since the nutritional benefits of consuming agricultural food resources can lead to greater reproductive success [65,66], elephants exploring the HDL may have access to fitness benefits unavailable or limited for elephants in the PA.

We found that there was a greater difference in the likelihoods of approaching and touching the objects between elephants near HDL and in the PA for those who investigated the novel objects quickly, while elephants who took longer to investigate were just as likely to approach and touch objects as those in the PA. Therefore, quick responses to novelty may be most beneficial to those elephants foraging in agricultural areas. Potentially, elephants can more successfully access agricultural resources if they approach novelty rapidly. For example, if a farmer installs a new fence, an elephant that is quick to approach may be able to explore and determine how to circumnavigate the novel barrier, as well as consume crops before being detected and driven away by the farmers who are often nearby patrolling their fields. Elephants who do approach and touch the objects, but are more hesitant and take longer to do so, behave similarly between the PA and HDL. Even with this interaction effect between location and latency measures, our results show that, overall, elephants near HDL were more likely to approach and touch novel objects than those in the PA.

It is important to note that elephants in this population have not been tracked continuously across the landscapes. We categorized elephants based on where they were tested (rather than where they predominantly live, which is unknown) to be conservative in our analyses. However, in this experiment, it is notable that we did not observe the same individuals in both HDL and PA locations, even across the different time periods of object installation. This might suggest that elephants with different behavioural traits spend more time in one type of landscape than another depending on the level of anthropogenic disturbance. However, our study sites do not cover all the agricultural areas bordering Salakpra, so it is possible that we missed the movement patterns of some elephants. Alternatively, elephants may flexibly adjust their behaviour to be more neophilic and exploratory as they move into HDL. While our study is limited in our ability to determine which of these explanations is supported by our results, our data demonstrate that elephants exhibit more neophilic and exploratory behaviour in environments in which there is greater human presence and disturbance.

Without a historical record of elephant behaviour in this landscape, it is impossible to determine whether elephants have become more neophilic as individuals spend more time foraging in HDL and are experiencing more novelty, or if those who were already more neophilic were attracted to HDL and spend time in these landscapes due to the fitness benefits of their neophilic behaviour. Given elephants’ long lifespans and the relatively rapid development of HDL within their lifetimes, it is unlikely that there has been enough evolutionary time for the differences in behaviour between landscapes to have evolved as an optimal response to novelty. Elephant foraging on agricultural resources has been documented for more than 50 years in Asia [65], but due to increasing natural habitat loss and fragmentation, more elephants are foraging in and moving through HDL (e.g. [67]). Greater neophilia in individuals may benefit the elephants’ use of these new landscapes, reflecting similar patterns as invasive bird species [22,23]. Future research could monitor changes in responses to novelty in populations over time to determine how neophilia and exploration may shift as landscapes change.

We did not find any significant effects of sex or age class on elephants’ attraction to or exploration of novel objects. We expected females to approach and interact with novel objects differently than males, given the potential risks novelty could pose to the offspring in the care of females; however, our results did not support this hypothesis. Male elephants are often assumed to take greater risks than females, particularly in the context of agricultural foraging [43,68], but this may vary by context. We also expected younger individuals to be more attracted to novelty and more exploratory than older individuals, as has been observed in primates, birds and in one study of Asian elephants [47,69–71]), although other research has demonstrated that older individuals approach novel objects first [72]. The lack of association that we observed in this study could mean that elephants do not vary in these behaviours based on age. Alternatively, our results may have been impacted by our limited sample size, which was skewed towards adults and subadults. We also did not find a significant effect of whether the subject was in a social group on the latency or duration measures in this study, indicating that social support did not influence the elephants’ behaviour around novelty overall. However, it would be more informative to evaluate this influence with a repeated measures study where individuals’ neophilia could be tested both when they were with a social partner and when alone. To collect these data in a wild setting would require a longer-term experiment to opportunistically expose the same individuals to novelty in different social settings. Further research with captive elephants, where social contexts could be controlled, would be beneficial to understanding the relationship between sociality and neophilia as well. Future research should also assess how neophilic behaviour in some individuals may affect group behaviour. It is possible that either social groups foraging in HDL are led by a few particularly neophilic individuals or all individuals in these groups exhibit greater neophilia than groups in the PA. Prior elephant visits also did not affect individuals’ behaviour, indicating that elephant odours on the object, which may have remained between object cleanings, were not skewing elephants’ responses towards the object.

In this study, we did not find consistent neophilia and exploratory measures for the elephants exposed to the two different novel objects. Therefore, we cannot conclude whether neophilia is a personality trait in this wild population. It is possible that our sample of only 19 elephants who were exposed to both objects was too small to show strong consistency, especially in the wild environment where there may have been many uncontrollable factors affecting elephant behaviour. We had hoped that more individuals would encounter both object types, but potentially due to elephant movement patterns, many individuals did not opportunistically encounter both objects in the time periods during which they were installed. Future research could increase the length of time during which stimuli are installed in the wild in order to observe more repeat visitors. Collecting data for elephants’ exposures to more than two novel stimuli across time could also lead to a more accurate measure of repeatability in the future [73]. It is also possible that certain characteristics of the brush made it less attractive to elephants, perhaps leading to elephants behaving less consistently towards the brush than towards the firehose object. In this study and in most novel object studies across species, objects are chosen based on their visible characteristics, which assumes that animals perceive novelty—and thus decide to interact with it—primarily using vision [15]. The texture of a novel object may also be a relevant stimulus once contact is made with it. However, for elephants, smell and sound are also highly relevant sensory modalities in their foraging and social decision-making processes [74], so it is possible that elephants would have more consistent reactions to novel stimuli presented in these sensory modalities.

Although responses to novelty seem to be consistent traits across many species [14], it is also possible that neophilia is not a personality trait for elephants and instead they behave flexibly towards novelty in different contexts. A study of zoo-housed African and Asian elephants’ responses to novelty also did not show consistent reactions between the three presented test stimuli [38]. While we have some concerns about the methodology of this study as it pertains to the types of stimuli used to test the novelty response, it is still interesting that both this study and our own did not find consistency in elephant responses across objects. Barret & Benson-Amram [38] also had a relatively small sample size (*n* = 15 elephants), so future research should include a greater sample of elephants and, if possible, include testing with more than two types of novel stimuli to better assess variation in elephants’ responses to novelty.

While studying wild elephants in their natural environment increases the ecological validity of the results, there are several limitations of conducting this novel object experiment in the wild. We attempted to account for many variables that could have affected elephant behaviour in analyses, but there were other variables beyond our control during novel object exposures. Some of these factors include an elephant’s sexual status, hunger or motivation, the type of social interactions occurring while an individual was within the 10 m perimeter of the object and other sensory stimuli that may have distracted from the novel objects, such as distant vocalizations or the urine and faeces of other elephants present in the area. These factors may contribute to the inconsistency we observed in elephant responses to the two objects. More longitudinal research across longer time spans is needed to determine whether neophilia and exploration are exhibited consistently as personality traits in Asian elephants and to assess the effect of personality on the decisions animals make as they navigate rapid human-driven change to their environments [29,30].

## 5. Conclusion

Overall, this study presents new evidence of patterns of individual animal variance in neophilia and novelty exploration across gradients of human disturbance from a new taxon. The results also have implications for applying knowledge about elephants’ attraction to novel objects in HDLs to conservation efforts. Specifically, information about how elephants vary in their responses to novelty could help guide the development of more effective methods of deterring elephants from agricultural fields. Elephant foraging on agricultural products is a major problem for the farmers in our study landscape in Thailand and across the Asian elephant’s range [28]. Many strategies currently used to deter elephants from crop fields are inconsistently effective, and understanding individual variation in behaviour could help target methods towards ‘problem’ elephants [29]. Also, identifying specific elephant behaviours associated with behavioural flexibility will help when predicting the elephants’ ability to adapt to continuing anthropogenic disturbance and environmental change [75].

Our findings with elephants may also help when predicting the behavioural patterns of other species increasingly competing with people for resources in HDLs. Human–wildlife conflict is a major conservation issue globally, and innovative solutions can contribute to the promotion of coexistence between humans and other species [76]. Further study of animals’ reactions to novelty in the wild may help in managing the negative interactions between humans and other animals by informing new mitigation strategies that consider the importance of research in behaviour, cognition and personality.

## Data Availability

The datasets supporting this article are available in the Dryad repository [77]. Supplementary material is available online [78].
